# Patient Satisfaction Among Opioid Use Disorder Treatment Sample in an Opioid Treatment Program: A Mixed Method Research Study

**DOI:** 10.3390/epidemiologia6040069

**Published:** 2025-10-30

**Authors:** Stanley Nkemjika

**Affiliations:** Department of Psychiatry and Human Behavior, Thomas Jefferson University, Philadelphia, PA 19107, USA; stanley.nkemjika@jefferson.edu

**Keywords:** treatment satisfaction, opioid use disorder, sociodemographic, social determinants of health

## Abstract

Background/Objectives: Patient satisfaction is increasingly recognized as a key indicator of the effectiveness of substance use disorder (SUD) treatment. While some studies suggest disparities in satisfaction across treatment settings, there remains limited research examining these differences, particularly among vulnerable populations. This study aimed to assess patient perceptions of satisfaction with opioid use treatment services and explore how demographic and socioeconomic factors influence these experiences. Methods: Conducted between 1 February and 31 March 2025, the study took place at a longstanding Opioid Treatment Program in Philadelphia. A total of 217 participants receiving treatment were recruited through convenience sampling during routine clinic visits. Data collection involved an electronically administered survey using the validated Client Satisfaction Questionnaire-8, with both quantitative and qualitative components. Quantitative data were analyzed using SAS 9.4, while qualitative responses underwent thematic analysis in Excel. Results: Findings revealed an average satisfaction score of 27.16, with employment status emerging as a significant predictor; employed individuals reported lower satisfaction (β = −1.118, *p* = 0.040), and race showed a marginal association. Qualitative analysis highlighted themes such as supportive staff, financial struggles, and personal growth. Conclusion: The results emphasize the need for equitable, culturally responsive treatment approaches that account for socioeconomic disparities in patient experience and care quality.

## 1. Introduction

In recent years, patient satisfaction has become a significant focal point in understanding the effectiveness of substance use disorder (SUD) treatments [1,2,3]. Research underscores the importance of patient satisfaction in improving both short- and long-term treatment outcomes, influencing retention rates, and promoting recovery [4]. Various studies have been conducted across different treatment settings, including residential addiction facilities and publicly funded SUD programs. For instance, one study highlighted the pivotal role of interpersonal relationships, trust, and individualized care in enhancing patient satisfaction in residential treatment settings [1]. The study found that approximately 70% of patients in residential programs were satisfied with their treatment, especially when a supportive and welcoming environment was emphasized [1]. Similarly, Carlson and Gabriel (2001) showed that patient satisfaction within publicly funded addiction treatment programs significantly predicted continued service use and positive recovery outcomes [5]. They found that satisfied individuals were 72% more likely to engage in follow-up care, reinforcing the notion that a positive treatment experience enhances long-term recovery success. These findings collectively emphasize the importance of treatment environments and interpersonal relationships in shaping patient satisfaction and influencing the course of recovery.

Another crucial determinant of patient satisfaction in substance use treatment is patient involvement in the treatment process. Studies have demonstrated that when patients are actively engaged in decision-making about their treatment goals, they are more likely to report higher satisfaction levels [6]. Andersson et al. (2017) found that patients who felt empowered in their treatment journey were 35% more likely to experience greater satisfaction compared to those who had less involvement in decisions about their care [1]. This participatory approach has been corroborated by Carlson and Gabriel (2001), who concluded that individuals who felt they had a voice in their treatment were more likely to experience positive recovery outcomes [5]. Furthermore, the physical and social aspects of the treatment environment also play a significant role in shaping patient satisfaction, as evidence suggests that patients’ perceptions of a safe and supportive environment are essential for fostering a sense of belonging and promoting recovery [7]. Hence, both patient-centered care models and the surrounding environment significantly impact patient satisfaction and treatment outcomes, highlighting the need for holistic treatment approaches that integrate both clinical care and social support.

Beyond the treatment environment and patient involvement, systemic factors such as access to services, waiting times, and treatment availability have been found to influence patient satisfaction [8]. Evidence in the literature shows that longer waiting times for mental health and substance use services were associated with lower satisfaction levels [9]. One study also highlighted significant regional disparities in satisfaction, with patients in higher-income areas reporting more positive experiences, likely due to better access to services [9]. Similarly, Kassaw et al. (2020), in their study of psychiatric patients in Ethiopia identified that effective communication between healthcare providers and patients was a critical factor contributing to satisfaction [10]. Patients who experienced shorter waiting times and felt respected by their healthcare providers were more likely to report higher satisfaction levels. These findings underscore the importance of addressing systemic barriers to care, such as service accessibility and wait times, to improve patient satisfaction and, by extension, treatment outcomes. Additionally, patients’ expectations of treatment have been shown to vary according to demographic factors like age, education, and socioeconomic status, with older and more educated individuals generally reporting higher satisfaction levels [10,11]. Despite the wealth of evidence linking patient satisfaction to improved treatment outcomes, significant challenges remain, particularly in lower-resource settings [12]. Notably, the lack of comprehensive post-treatment care and rigid treatment structures have been identified as major contributors to dissatisfaction and poor retention rates [13]. Inadequate post-treatment support leaves many patients feeling unprepared for the challenges of long-term recovery. Similarly, Harris et al. (2024) found that stigma surrounding SUD and mental health issues was a substantial barrier to satisfaction in certain regions [9]. These stigmatizing environments can undermine recovery efforts, as patients may feel ashamed or unsupported in their treatment journeys. These studies suggest that a more holistic and flexible approach, which addresses not only the clinical but also the socio-environmental aspects of care, is essential to improving patient satisfaction and ensuring better long-term recovery outcomes.

Irrespective of the growing body of research on patient satisfaction in SUD treatment, significant gaps remain, both globally and within the American population. Additionally, there is a notable absence of mixed-methods research examining treatment satisfaction among low-income and racial minority patients receiving care in opioid treatment programs. Many studies primarily focus on specific treatment settings, such as residential or outpatient facilities, without considering the intersectionality of diverse patient populations, including age, ethnicity, socio-economic status, and geographical location [2,4,8]. Moreover, while patient satisfaction is widely recognized as a predictor of treatment engagement and recovery, its long-term impact on public health outcomes, particularly in marginalized groups, remains underexplored. In the U.S., where SUD disproportionately affects vulnerable populations, such as low-income communities and people of color [14], there is a pressing need for research that investigates how systemic factors like accessibility, stigma, and cultural competency within treatment programs affect patient satisfaction. Addressing these gaps is crucial for fostering positive social change, as improving patient satisfaction can enhance treatment retention, reduce healthcare disparities, and ultimately contribute to better recovery outcomes. This has significant implications for public health practice, where a more patient-centered, equitable approach can help mitigate the social and economic burden of SUD, leading to stronger, healthier communities. Accordingly, this study aims to assess patients’ perceptions of satisfaction with their treatment outcomes of an opioid use disorder (OUD) sample, with the goal of informing more patient-centered and equitable approaches to SUD care. Hence, the objective of this study is to assess patients’ perceptions of satisfaction with their treatment outcomes among individuals with OUD.

## 2. Materials and Methods

### 2.1. Setting

This study was conducted at a longstanding Opioid Treatment Program (OTP) located in Philadelphia, Pennsylvania USA, between 1 February and 31 March 2025. The selected treatment program is among the oldest in the region and is situated in a city that has been notably impacted by the opioid overdose epidemic and broader SUD challenges. The program provides pharmacological treatment for OUD with methadone and buprenorphine, and also offers treatment for co-occurring substance use and psychiatric disorders using a wide range of pharmacological and non-pharmacological modalities. The patient population primarily comprises residents of the Philadelphia metropolitan area, though a subset of participants travel from suburban regions or neighboring Southern New Jersey. The program serves a demographically diverse cohort, largely composed of individuals from minority and socioeconomically disadvantaged backgrounds. Convenience sampling was employed in the selection of this program due to its accessibility and relevance to the study objectives. Data collection was timed to coincide with routine clinic visits and scheduled appointments, primarily during weekdays, to maximize participation and reduce disruption to clinical operations. A total of 217 participants were enrolled in the study. Eligibility was based on current engagement with treatment services at the clinic during the data collection period.

### 2.2. Ethical Consideration

Prior to participation, all respondents were provided with detailed information regarding the study objectives, procedures, and potential risks and benefits. There was no exclusion criteria for participation in the study as it was open to all patients receiving treatment in this OTP. However, participants must have been receiving treatment from the facility for more than a day (>24 h). Emphasis was placed on voluntary participation, and participants were explicitly informed of their right to withdraw from the study at any stage without jeopardizing their ongoing treatment. Ethical considerations were rigorously upheld throughout the study, with data confidentiality maintained in accordance with institutional guidelines and ethical standards for research involving human subjects. Participants were informed of the study’s purpose, procedures, and their rights, including the voluntary nature of participation and the freedom to withdraw at any time without consequence. The ethical conduct of the study was ensured through approval by the Ethics Committee and Institutional Review Board of Thomas Jefferson University (Protocol No. iRISID-2024-1252) approved 27 September 2024), with strict adherence to principles of confidentiality and data security.

### 2.3. Data Collection

Data collection utilized a mixed-methods concept focused on assessing patient-perceived satisfaction with treatment services, alongside relevant sociodemographic variables such as age, gender, and employment status. The primary data collection instrument was an interviewer-administered questionnaire based on the Service Evaluation Questionnaire, which was itself adapted and refined from the established Client Satisfaction Questionnaire (CSQ) templates. The widely used CSQ-8 instrument was employed to comprehensively capture patient satisfaction across multiple dimensions of care. Additionally, participants were encouraged to provide subjective perceptions of the treatment they had been receiving. Participants completed the survey electronically, using either a direct web link or a QR code that routed them to a secure domain within the Qualtrics platform. The survey was self-completed by participants using their personal cell phone or an electronic tablet device. This method facilitated efficient data capture while maintaining participant privacy and autonomy. Researchers were available to clarify any ambiguities and respond to participant inquiries during the data collection process, thereby ensuring the accuracy and completeness of responses. The study instruments were tailored to reflect the unique characteristics of the clinic’s patient population, which primarily comprises vulnerable, minority individuals of lower socioeconomic status.

### 2.4. Measures

The questionnaire was designed to collect baseline demographic attributes, length of stay in the OTP, satisfaction questionnaire and open-ended satisfaction question to assess the qualitative experience, “what is your general impression and perception of the services and treatment you have been receiving in this facility? How satisfied are you with the support and resources provided to you?”. The open-ended question was included to supplement and provide context to the quantitative analysis. Notably, the primary outcome measure of this study was patient-perceived satisfaction with SUD treatment services. This outcome was assessed using the Client Satisfaction Questionnaire (CSQ), specifically the CSQ-8 instrument which has a high internal reliability score (Cronbach’s alpha = 0.92–0.93). The CSQ-8 is a concise and widely validated tool comprising eight items that evaluate various aspects of service satisfaction, including overall contentment with care, perceived quality of services received, and the likelihood of recommending the clinic to others. These instruments provided robust measures for evaluating the effectiveness of service delivery from the patient’s perspective. Independent variables included a range of sociodemographic factors, such as age, gender, employment status, and the specific SUD for which the patient was receiving treatment. These variables were selected based on their relevance to the research question and their documented association with treatment outcomes in existing literature. The use of validated satisfaction measures enabled the study to generate meaningful insights into patient experiences and perceptions of care quality within the context of an Opioid Treatment Program. The CSQ-8 provided a comprehensive framework for assessing service quality, thus equipping healthcare providers and policymakers with actionable insights to improve patient-centered care. The application of this standardized instrument aligns with best practices in health services research and supports the comparability of findings across similar studies.

### 2.5. Data Analysis

Data entry commenced immediately upon survey completion, with responses captured in real time through the Qualtrics platform. Collected data were exported as CSV and Excel files for subsequent analysis using SAS version 9.4 statistical software. Initial analyses comprised descriptive statistics to summarize the sociodemographic characteristics of the sample, presenting frequencies for categorical variables and means with standard deviations for continuous variables. This approach facilitated a clear understanding of the participant profile and baseline characteristics. At the univariate level, simple descriptive analyses were conducted to estimate the prevalence of key variables of interest. Variables such as age, gender, employment status, and primary substance of use were examined to establish foundational insights into the study population. The relationships between these independent variables and patient satisfaction outcomes were then explored at the bivariate level using chi-square tests to assess associations for categorical variables. Variables demonstrating statistical significance at the bivariate level (*p* ≤ 0.05), particularly age, were further analyzed using multivariate logistic regression models. This advanced analysis enabled the identification of factors independently associated with patient-perceived satisfaction, controlling for potential confounding variables. Results from the multivariate models were interpreted in the context of existing literature, allowing for the evaluation of plausible explanations and the generation of evidence-based recommendations for practice and policy enhancement. Using the 2021 Microsoft Excel tool, qualitative thematic analyses were conducted based on the responses from participants in relation to final model.

## 3. Results

### 3.1. Demographic Characteristics

Of the 217 participants that completed the survey, 42 provided qualitative responses to the open-ended question. Table 1 shows that the study sample had a mean age of 46.52 years (SD = 10.37). The average duration of treatment was 86.44 months (SD = 84.18). The mean score on the Client Satisfaction Questionnaire-8 (CSQ-8), a validated measure of treatment satisfaction, was 27.16 (SD = 5.04). In terms of racial and ethnic composition, the majority of participants identified as non-Hispanic White (71.21%), followed by non-Hispanic Black (19.19%), Hispanic (5.05%), biracial (2.53%), Asian (0.51%), and other racial groups (1.52%). Gender distribution was evenly split, with 50% identifying as male and 50% as female. Regarding educational attainment, 27.78% of participants had fewer than 11 years of education, 41.92% had completed 12 years of education (high school diploma), 9.60% reported post-high school vocational or technical training, 13.13% had some college education, 6.57% were college graduates, and 1.01% held a postgraduate degree. Employment status indicated that 48.99% of participants were unemployed, 12.12% were retired, 6.06% were employed part-time, 1.82% were employed full-time, and 1.01% were students. In terms of marital status, 72.22% identified as single, 11.11% as married, 5.05% as widowed, and 1.62% as divorced. Most participants (73.23%) reported a religious affiliation, while 26.77% reported no religious identification. Regarding annual income, 71.72% reported earnings below $25,000, 21.21% earned between $25,000 and $50,000, 5.56% earned between $50,000 and $75,000, and 1.52% reported income exceeding $75,000. With respect to medication-assisted treatment, 95.95% of participants were receiving methadone, 4.04% were on buprenorphine, and 1.01% were using other medications.

### 3.2. Bivariate Correlations

Spearman’s rho correlations were conducted to examine associations between demographic variables and perceived satisfaction (CSQ-8 scores). Age was negatively correlated with satisfaction (ρ = −0.136, *p* = 0.061), though this did not reach statistical significance. Gender was not significantly correlated with satisfaction (ρ = 0.043, *p* = 0.546). Educational level showed a non-significant negative correlation (ρ = −0.126, *p* = 0.077), while race was significantly negatively correlated with satisfaction (ρ = −0.147, *p* = 0.041). Income was positively associated with satisfaction (ρ = 0.141, *p* = 0.049), and employment status was significantly negatively correlated (ρ = −0.170, *p* = 0.017). Religious affiliation (ρ = −0.110, *p* = 0.124), marital status (ρ = −0.036, *p* = 0.620), and length of treatment (ρ = −0.008, *p* = 0.907) did not show significant associations with satisfaction. See Table 2 for further details.

### 3.3. Regression Analysis

Simple linear regression analyses were conducted to further examine the association between individual predictors and patient satisfaction scores (Table 3). Race was significantly associated with satisfaction, with a parameter estimate of −1.022 (SE = 0.497, *p* = 0.041), indicating lower satisfaction among racial minority groups. Income was also a significant predictor of satisfaction (estimate = 1.162, SE = 0.578, *p* = 0.046), with higher income levels associated with greater satisfaction. Employment status was significantly negatively associated with satisfaction (estimate = −1.284, SE = 0.533, *p* = 0.017), suggesting that unemployed individuals reported higher satisfaction. Other demographic variables did not exhibit statistically significant relationships with satisfaction. Table 4 presents the results of the final multiple linear regression model evaluating predictors of patient-perceived satisfaction following SUD treatment. The model included race and employment status as independent variables. Race was negatively associated with satisfaction, with a parameter estimate of −0.906 (SE = 0.496), though this association did not reach conventional levels of statistical significance (*p* = 0.069). Employment status remained a significant predictor, with a parameter estimate of −1.118 (SE = 0.539, *p* = 0.040), indicating that individuals who were employed reported lower satisfaction scores. The model intercept was estimated at 30.365 (SE = 1.160, *p* < 0.001). The overall model demonstrated a modest fit, with an R^2^ value of 0.043. The model was statistically significant, F(2, 184) = 4.30, *p* = 0.015. See Table 3 for more details.

### 3.4. Qualitative Thematic Analysis of Reported Satisfaction Among Participants

Table 5 shows the qualitative thematic interpretations of participants’ reported perceived satisfaction. The themes include:

Overall Positive Perception: Despite the economic difficulties and significant levels of unemployment, many respondents expressed an overall positive impression of the program. The cohort consisted of a large majority (67%) of non-Hispanic White (NHW) as well as non-Hispanic Black (NHB) (33%) participants. Eighty-nine percent of participants had an annual income of less than $25,000. Of these, two-thirds were unemployed (67%), with the remainder being employed (22%) or retired/students (11%). This group’s overall positivity suggests that elements such as supportive staff and a caring program environment may significantly influence participant attitudes even in the face of socioeconomic adversity.

Supportive Staff Encounters: Across racial and employment backgrounds, participants consistently emphasized the importance of supportive and empathetic staff. This theme encompassed NHW and NHB populations, primarily from low-income demographics, including unemployed, employed, retired, and students. These findings suggest that relationships with culturally responsive and kind staff are a universally valued aspect of the program experience.

Challenges: Many participants discussed their financial struggles and background factors that made success difficult. This group was also predominantly NHW (67%), though others were NHB or from other racial backgrounds. Eighty-three percent reported earning less than $25,000 annually, and unemployment was evenly split between employed and unemployed individuals. These findings suggest that income, more than employment status alone, is critical to understanding participants’ barriers.

Personal Growth: Reports of personal growth were most often shared by NHB participants (75%), all of whom reported incomes below $25,000. Most were retired or students (75%), while 25% were unemployed. These accounts show how personal change is possible, even amid financial adversity, especially when participants fully engage with the program.

Staff Dissatisfaction: Notably, complaints about staff interaction were raised mostly by participants who were relatively economically secure. The group was evenly divided in race between NHW and NHB (50% each) and predominantly earned between $25,000 and $50,000 annually (75%). More than three quarters (75%) were working, with the rest being students or retired. This suggests that participants with higher incomes or more stable employment may have higher expectations of program staff.

Positive Personal Completion Outcome: In one notable outlier whose race is other than NHW or NHB listed their annual income as over $50,000. This person was fully employed, and had a positive personal outcome after completing the program. This particular instance, although unique, may suggest a possible association between economic robustness and positive outcomes that like further data to help alleviate some type of relation.

Cross-Theme Insights: Several consistent patterns emerged across themes. There were several commonalities among the groups, including economic hardship: A large percentage of those in each category had income less than $25,000. However, the employed reported positive program experiences from their employment status alone was not the single driving factor of their satisfaction. Finally, NHB participants often played a prominent role across both success and challenge stories, underscoring the complex role race plays in how respondents experienced and evaluated the program.

## 4. Discussion

This mixed-methods research, a cross-sectional study explored patient-perceived satisfaction among a socioeconomically vulnerable population undergoing SUD treatment, with a particular focus on demographic predictors. The average CSQ-8 satisfaction score (27.16) indicated a generally favorable perception of treatment, yet substantial disparities were observed based on race, income, and employment status. The demographic composition of the sample, largely NHW, predominantly low-income, and nearly 49% unemployed, reflects the broader socioeconomic marginalization common among individuals with SUDs [15]. Notably, the high proportion of participants receiving methadone (95.95%) aligns with existing literature, which indicates methadone is frequently used in long-term maintenance therapy for OUD, especially in low-resource settings, and it is frequently designated as a first-line pharmacologic intervention [16]. Additionally, this high proportion of methadone use in this OTP is mainly people who do not want or who have failed treatment with buprenorphine. The perceived disparities in satisfaction, based on the qualitative results suggest inequitable treatment experiences at the structural and institutional level despite including participants across race, education, and employment lines [17]. These findings highlight the importance of tailoring addiction services to patients based on demographic information.

Our study findings in terms of correlational analysis indicated significant associations between race, income, employment status, and perceived satisfaction. More specifically, identification with a racial minority group was negatively correlated with satisfaction, meaning racial minority groups were less likely to report positive treatment experiences compared to some of their peers. This supports longstanding concerns in the literature about racial disparities in health outcomes and experiences due to structural inequities, provider bias, and cultural disconnects in care delivery [18]. Income was positively associated with satisfaction, suggesting that patients with more economic stability perceived better treatment outcomes, perhaps due to enhanced access to supportive services and fewer financial stressors during recovery [19]. Moreover, employment status was negatively and significantly correlated with satisfaction, such that employed participants reported lower treatment satisfaction. These associations indicate that social determinants of health, including race, economic hardship, and unemployment, have a considerable influence on patient understanding and engagement with SUD treatment [20].

However, the unique nature of our study posits that patients with jobs were less likely to endorse treatment satisfaction. On average, employed individuals report satisfaction scores 1.118 points lower than unemployed individuals, and this effect is statistically significant based on this study. This finding may reflect systemic barriers that employed individuals face in accessing and engaging with treatment services. One plausible explanation for this disparity lies in the differing expectations and life demands between employed and unemployed individuals. These expectations, if unmet, may result in more critical evaluations of treatment effectiveness [2]. The role of expectation setting is well-documented in the patient satisfaction literature, where the disconnect between anticipated and actual care experiences is a key determinant of perceived dissatisfaction [21]. Employed individuals may prioritize functionality and rapid recovery, aligning treatment success more closely with their ability to maintain productivity and minimize disruptions to their professional life. For example, rigid work schedules may conflict with treatment appointments, and fear of stigma or job-related consequences could reduce comfort and openness in care settings [22]. Additionally, employed individuals may perceive treatment as less accessible or less flexible, leading to decreased satisfaction with service delivery [23]. In contrast, unemployed individuals may experience fewer external pressures and may perceive treatment as a more integral part of their daily routine, leading to greater appreciation of its benefits. These psychosocial dynamics underscore the need for further qualitative and mixed-methods research to explore how employment status interacts with treatment expectations, contextual stressors, and care delivery. Such investigations are essential for informing more equitable, patient-centered models of OUD care that are sensitive to the realities of diverse life circumstances. From a theoretical standpoint, these results support the social determinants of health framework, which posits that employment though typically seen as protective can, under certain conditions, pose challenges to accessing equitable care [24]. In the context of OUD treatment, being employed may introduce constraints that may diminish overall patient treatment satisfaction. Hence, highlighting the need for more work-friendly treatment models, such as extended hours, telehealth, or employer education programs.

The regression models from our study further validated these associations, with race, income, and employment status emerging as significant predictors of satisfaction in the simple models. While race as a variable remained negatively associated with satisfaction in the multiple linear regression model, its *p*-value slightly exceeded the conventional threshold for significance (*p* = 0.069), likely due to reduced statistical power when controlling for multiple variables. But qualitative thematic findings showed that individual growth was often identified as a theme among NHB participants. However, employment status remained a significant predictor in the final model (*p* = 0.040), corroborating our hypothesis that structural exclusion from the labor market can adversely affect patient perceptions of care [25]. Income was an important predictor in the simple model but did not remain significant in the multiple regression models, likely due to its correlation with employment. The final regression model accounted for 4.3% of the variance in satisfaction scores (R^2^ = 0.043), suggesting that while demographic factors play a role, other psychosocial or treatment-specific variables (e.g., counselor rapport, perceived stigma, or access to wraparound services) also contribute to perceived satisfaction [26]. These results reflect the complexity of satisfaction as an outcome metric and call for multidimensional frameworks when evaluating patient experiences in SUD treatment settings.

Thematic analysis of participant feedback provided qualitative insights into the factors influencing satisfaction in SUD treatment among vulnerable populations. Participants specifically highlighted how compassionate and culturally responsive staff were central to their treatment experience, particularly for individuals from low-income backgrounds. This finding is supported by previous literature emphasizing the importance of staff competence and user involvement for treatment outcomes. For example, a study by Yang et al. demonstrated that trust and rapport with the counselor form key facilitators of treatment engagement, especially in clients completing inpatient SUD programs [27]. In contrast, those with higher income or more stable work reported dissatisfaction with staff interactions, indicating that those with higher expectations may be more critical of service quality. This is similar to the results from the literature; clients whose treatment expectations have not been met often report being less satisfied than those with a lower level of expectation [2]. Moreover, individual growth was often identified during interviews among NHB participants. This growth suggests that dairying and other internal knowledge exchanges can lead to positive outcomes despite pitfalls or barriers related to the struggling economy. This supports the literature indicating that attendance in mutual-help groups and excellent retention in treatment are facilitators of substance use improvements among NHB clients [2,25]. These qualitative themes further elucidate the quantitative real-world findings and suggest areas for targeted enhancement in treatment delivery. Treatment programs can invest their time and resources more effectively by working directly with client-facing staff to increase the quality of interactions (with all clients, but especially those with higher expectations) to create supportive and empathetic environments, thus improving their overall satisfaction and response to treatment. In addition, regarding NHB participants specifically, integrating principles of self-development and internal motivation in care can be beneficial as this group may have different needs than their NHW counterparts. These strategies may help improve treatment outcomes and satisfaction among vulnerable populations in SUD treatment programs.

### 4.1. Implications for Public Health Practice, Policy, Research, and Positive Social Change

Our study findings have important implications for public health practice, research, and policy. From a policy perspective, the finding of lower satisfaction among racial minorities and unemployed individuals indicates a critical opportunity to prioritize equity and cultural competence in SUD treatment programs. Employment support services, mental healthcare, and culturally meaningful interventions should be integrated into public health systems to meet the diverse needs of vulnerable populations [28]. Notably, the results from our study serve as an impetus for a broader investigation into patient–provider dynamics, systemic discrimination, and the role of psychosocial support in mediating treatment experiences from a research perspective. Theoretically, these results are consistent with the social determinants of health framework that highlights the role of non-medical factors such as economic conditions, race, and social policies in shaping health outcomes [29]. From a policy perspective, this study supports designing quality metrics to raise the quality of SUD treatment in a way that is sensitive to demographic inequities. More broadly speaking, these findings advance social change by illuminating structural inequities that threaten the efficacy and significance of addiction treatment. This more inclusive and socially responsive healthcare system has potentially long-lasting implications in promoting reforms that support the recovery and dignity of all individuals impacted by these disorders. The implications of this study extend to positive social change through increasing awareness of how an individualized approach to SUD treatment that caters to special populations may lead to greater success in the management of SUDs. It provides opportunities for shaping equity-oriented interventions based on determining characteristics of patient satisfaction. Improving patient satisfaction can improve engagement in treatment, reduce dropout rates, and improve long-term health outcomes, creating healthier communities while also reducing the societal burden of substance use disorders.

### 4.2. Strengths and Limitations

Among the key strengths of this study is its focus on a marginalized and often understudied population, specifically individuals undergoing long-term medication-assisted treatment (MAT) for SUD. The findings are strengthened by the use of the CSQ-8, which is a validated instrument for measuring satisfaction. Additionally, the rich demographic information collected and the qualitative thematic analysis conducted during the study allow for an in-depth examination of intersectional vulnerabilities such as race, income, and employment status. However, some important limitations should be noted. Due to the cross-sectional nature, this study cannot infer cause–effect relationships between demographic characteristics and outcome measures of satisfaction. Self-reported data may be influenced by recall or social desirability bias, particularly in sensitive areas like employment and income. Additionally, the generalizability of the results may be limited, given the overwhelmingly methadone-based treatment modality, which may not reflect the experiences of individuals on alternative medications like buprenorphine or naltrexone. Furthermore, the modest R^2^ value of the regression model suggests that future research should incorporate broader psychosocial and environmental variables to capture the satisfaction determinants better.

## 5. Conclusions

This study finds notable differences in patient-reported satisfaction with SUD treatment among a vulnerable population, particularly influenced by race, income, and employment status. These results highlight the need for treatment approaches that are equitable, culturally responsive, and sensitive to the challenges faced by patients from varying socioeconomic backgrounds, given that patient experience and retention are significantly linked to successful treatment outcomes. Despite moderate satisfaction levels overall, structural barriers remain a significant determinant of the perceived quality of care for the most disadvantaged groups. It is necessary to tackle these disparities via integrated support services and policy reform to achieve better treatment outcomes. Future research should explore additional psychosocial and institutional factors influencing satisfaction to inform more holistic, patient-centered care strategies.

## Figures and Tables

**Table 1:** Sociodemographic attributes of SUD treatment population.

Demographic Characteristics	Frequency (N)	Percentage/ (Mean ± SD)
Age (years)	192	46.52 ± 10.37
Length of Treatment (Months)	196	86.44 ± 84.18
CSQ-8 Satisfaction Score	197	27.16 ± 5.04
Race		
NHW	141	71.21
NHB	38	19.19
Asian	1	0.51
Hisp	10	5.05
Bi-Racial	5	2.53
Others	3	1.52
Gender		
Male	99	50.00
Female	99	50.00
Education		
<11 years	55	27.78
12 Years or completed high school	83	41.92
Post High School Training	19	9.60
Some College	26	13.13
College Graduate	13	6.57
Postgraduate	2	1.01
Employment status		
Full-time	63	31.82
Part time	12	6.06
Unemployed	97	48.99
Retired	24	12.12
Student	2	1.01
Marital Status		
Single	143	72.22
Married	22	11.11
Divorced	23	11.62
Widow	10	5.05
Religion Identification		
Yes	145	73.23
No	53	26.77
Income		
<$25 K	142	71.72
$25 k–$50 K	42	21.21
$50 K–$75 K	11	5.56
>$75 K	3	1.52
Medications		
Methadone	188	94.95
Buprenorphine	8	4.04
Others	2	1.01

**Table 2:** Spearman Correlation based on Patient perceived Satisfaction.

CSQ-8		
Variables	r	*p*
Age in years	−0.136	0.061
Gender	0.043	0.546
Education	−0.126	0.077
Race	−0.147	0.041 *
Income	0.141	0.049 *
Religion Identification	−0.110	0.124
Employment Status	−0.170	0.017 *
Marital Status	−0.036	0.620
Length in Treatment	−0.008	0.907

* *p* < 0.05.

**Table 3:** Simple Regression Analysis based on Patient Perceived Satisfaction.

	Age	Gender	Education	Race	Income	Religion	Employment	Marital	LOT
	*Beta*								
Intercept	30.23 *	26.503 *	28.360 *	28.567 *	25.582 *	28.741 *	29.400 *	27.460 *	27.203 *
Age	−0.064								
Gender		0.434							
Education			−0.523						
Race				−1.022 *					
Income					1.162 *				
Religion						−1.248			
Employment							−1.284 *		
Marital								−0.303	
Length of Rx									−0.001

Key-* denotes *p* < 0.05; Race-se = 0.497, *p*-value = 0.041; Employment-se = 0.533, *p*-value = 0.017; Income-se = 1.162, *p*-value = 0.046.

**Table 4:** Final Regression model of Patient perceived Satisfaction.

	Est (b)	SE	*p*-Value
Intercept	30.365	1.160	<0.001
Race	−0.906	0.496	0.069
Employment	−1.118	0.539	0.040

R^2^ = 0.043; F(2, 184) = 4.30, *p* = 0.015.

**Table 5:** Qualitative Thematic Analysis based on Patient Satisfaction.

Theme	Race	Income	Employ
Financial Challenges	Non-Hispanic White: 67.0% Non-Hispanic Black: 33.0%	<$25 K: 83.3% $25 K–$50 K’: 16.7%	Employed: 50% Unemployed’:50%%
Overall Positive Perception	Non-Hispanic White: 88.9% Non-Hispanic Black: 11.1%	<$25 K’: 88.9% $25 K–$50 K’: 11.1%	Unemployed: 66.7% Employed’: 22.2% Retired/Student: 11.1%
Personal Growth	Non-Hispanic Black: 75.0% Non-Hispanic White: 25.0%	<$25 K’: 100.0%	Retired/Student: 75.0% Unemployed: 25.0%
Positive Personal Outcome Completion	Other: 100%	More than 50 k: 100.0%	Employed: 100.0%
Staff Dissatisfaction	Non-Hispanic White: 50.0% Non-Hispanic Black: 25.0% Other’: 25.0%	$25 K–$50 K: 75.0% <$25 K’: 25.0%	Employed: 75.0% Retired/Student: 25.0%
Supportive Staff Encounters	Non-Hispanic White: 78.9% Non-Hispanic Black: 15.8% Other: 5.3%	<$25 K: 78.9% ‘$25 K–$50 K’: 21.1%	Unemployed: 47.4% Employed: 36.8% Retired/Student: 15.8%

## Data Availability

The data that support the findings of this study are available from the corresponding author upon reasonable request due to privacy concerns.
